# Chronic Cystitis—Perineal Cystotomy

**Published:** 1872-06

**Authors:** Robert Battey

**Affiliations:** Rome, Ga.


### CHRONIC CYSTITIS—PERINEAL CYSTOTOMY.

By Robert Battey, M. D., Rome, Ga. *Read Before the Georgia Medical Association.*

Mr. P., aged fifty-nine, a robust and healthy man, consulted me in 1866 for anal fistula, and was submitted to operation on the 17th of that month, by *ecrasement*, after the manner of Chassaignac, followed by a prompt and perfect cure.

From this time he enjoyed uninterrupted health until he contracted a specific purulent urethral discharge in October, 1867, which readily yielded to treatment, and all seemed to go on well. In the Spring of 1868 a small abscess formed in the cellular tissue underlying the middle of the urethra, with little or no complaint of urethral obstruction, and but little urethral irritation. The abscess healed soundly and in a short time, and still all seemed to go on well with him.

On the 9th of September, 1868, I found him complaining greatly with sub-acute cystitis, but his symptoms did not indicate stone. For some months he was submitted to treatment by the usual remedies varied from time to time, with varying results; sometimes better, sometimes worse, but no substantial relief. He was twice sounded with negative results as to stone.

On the 11th of April, 1869, his condition becoming more and more serious, I sounded again, very carefully under chloroform, and confirmed my diagnosis by that of Dr. W. D. Hoyt, who was kind enough to assist me. There was no stone.

He was now suffering most intensely, voiding urine every hour or two hours, with excruciating pain, which was with difficulty rendered at all tolerable. There was much discharge of vesical mucus, but no blood. Efforts were made to have the bladder properly washed out, but as the patient resided at a distance in the country and could not command skillful nursing, it was not satisfactorily accomplished.

Thus the case wore on with lengthening days and anxious nights, until it seemed indispensible that the bladder and the patient must have *rest*, or the period of dissolution could not be many weeks distant. But how was this rest—local rest to the worn-out bladder, and general rest to the well-nigh exhausted invalid—to be obtained? Evidently the functions of the bladder must for a time be suspended, and its membrane systematically cleansed and soothed. To fill these indications satisfactorily, the practical suggestion of Dr. Barker, of New York, approved by Professor Gross, of Philadelphia, of opening the neck of the bladder by lateral perineal incision, was adopted, as presenting on the whole the best hope of eventual cure. This proceeding too, it was considered, would afford an opportunity for thorough digital exploration of the interior of the viscus, and for the detection of any chance calculus which may have been undiscovered in sounding.

On the 3d of June this determination was put in execution. The usual lateral incision was made under chloroform. There was no hemorrhage, nor any untoward event. There was neither calculus nor incrustation. The bladder was contracted, thickened, and about the neck and trigone it was ulcerated. A good sized gumelastic tube was secured in the wound by a stitch through the tissues tied around the projecting portion. The urine dripped continuously through the tube as it was delivered from the ureters.

June 4.—Pulse 84, tongue a little dry, doing well. Washed out the bladder well with syringe and catheter through the gum-elastic tube.

June 6.—Pulse 72, tongue clean and moist, relishes his meals, is very comfortable, cheerful, and hopeful; is quite enthusiastic over the operation; says he has not suffered as much, put it all together, since the the operation, as he did in any twenty minutes before. Washed out the bladder, and prescribed tincture of iron; the syringing to be repeated thrice daily.

June 21.—Pulse 80, appetite good, mind cheerful, wound healthy; the tube had been forced out of the bladder, replaced it, and washed out the wound; clear urine, with a few shreds of mucus; no pus.

June 13.—Complains of the stitch which holds the tube; says the tube comes out when he goes to stool and has to be replaced, which gives him pain. Clipped the stitch, and secured the tube with adhesive plaster.

June 15. Doing well; pulse 80, apetite good, wound healthy; little or no pain or mucus from the bladder.

June 19.—Pulse 80, and good; complains a little of vesical pain. He has slept well, and required no anodyne since the dose of opium which followed the operation. Will take a dose of McMunn's Elixer at bed-time; continue iron.

June 26.—General condition good; has had sore on sacrum from neglect of cleanliness. Ordered dressing of adhesive plaster, and better attention.

July 3.—He is very comfortable; bedsore healed. Directed wash for bladder, 20 grains sulphate of zinc to a pint of water.

July 9.—Mr. P. "thinks he is better;" complains of the weak zinc injection smarting and burning for some time after it is used. His general state is very good; wound is healed firmly round the rubber tube; water thrown in by the tube escapes freely by the urethra.

July 16.—Eats well; gaining strength; walks about his room; still complains of the zinc injection. Removed the rubber tube and found it thickly incrusted with a ring of deposited phosphates.

July 23.—Not so well for two days; pulse 80; appetite poor; bowels constipated; complains of soreness in the region of the kidneys; urine does not dribble, but is passed periodically, both by urethra and perineum, and with considerable pain at each micturition; the mucous membranes are becoming quite pallid. Ordered sinapisms to the back; two drachms of the tincture of iron daily; zinc discontinued in the wash for the bladder and morphine substituted.

July 28.—Pulse 80; more feeble; skin cool and rather clammy; appetite bad; complains of pain in the right kidney; uses opium quite freely, and the tincture of iron as much as his stomach will bear.

August 1.—Pulse 89; is much better than when I last saw him; sat in a chair, and talked with me very hopefully of his condition. He voided urine *per urethrum* in my presence, and said it gave him little or no pain. The urine is good; there is no pain in the kidney.

August 7.—Seems to be better, but his bladder is again secreting the ropy mucus. Ordered copaiba. 30 drops daily.

August 15.—Pulse 80; strength and appetite declining; considerable pain and tenderness about the right kidney. He complains much of pain in the right hip, knee, and especially the *heel*. The urine is more loaded with mucus and pus.

August 30.—Sits up; is stronger; has good appetite; voids a good deal less mucus. He uses a wash of carbolic acid for the bladder with apparent benefit, and continues tincture of iron.

September 8.—Condition of the bladder much improved; the wash of carbolic acid caused swelling of testicle and cord, but it is now subsiding under the local use of tincture of iodine.

September 19.—Mr. P. seems bright; found him sitting up reading his Bible; quite improved since my last visit; has good appetite and gaining strength. There is an occasional mucous discharge from the bladder; pain moderate; sleeps three hours at night without passing urine; there is no leakage in the perineum.

September 30.—Mr. P. visited me at my office, and spent the day in town quite comfortably. Says he is improving very rapidly under the use of a vesical wash of chlorate potassa two or three times a day. He finds it very soothing and comforting to him.

October 6.—Called at my office in my absence.

October 16.—In my office; is "quite comfortable in every way; rides about everywhere." He suffers very little pain, retaining urine from two to three hours; there is no discharge of vesical mucus; uses the chlorate injection twice daily. He has acquired the power of voiding urine either by the urethra or by the perineal opening at will. This option he esteems of special value, and would not part with the perineal fistula for any consideration.

October 30.—Has taken cold; return of orchitis, and pain in the bladder.

From this relapse he soon rallied and continued to transact his business in town, and to consult with me at my office for many months. I did not have cause to visit him again at his home until the 23d of September in the following year (1870). His physical sufferings were now returning upon him as aforetime, and the beginning of the end seemed near at hand. The vesical catarrh returned with redoubled energy, and with decided suppuration.

The disease hurried forward in its downward course, and terminated in death on the 28th of November, 1870. Unfortunately, I was not informed of the decease in time for *post mortem.* This I specially regretted, for I had long previously arranged with the patient himself for this inspection in the event of a fatal termination.

